# Early Squamous Cell Carcinoma With Perineural Invasion: A Prospective Study Examining Anatomic Site, Tumor Surface Diameter, Invasion Depth, and Grade of Differentiation in 1,772 Consecutive Cases

**DOI:** 10.5826/dpc.1003a59

**Published:** 2020-06-29

**Authors:** John H. Pyne, Esther Myint, Simon P. Clark, Maddie Gorji, Ruihang Hou

**Affiliations:** 1 Prince of Wales Clinical School, University of New South Wales, Sydney, Australia

**Keywords:** squamous cell carcinoma, perineural invasion, grade of differentiation, invasion depth, anatomic site

## Abstract

**Background:**

Squamous cell carcinoma (SCC) may present with perineural invasion (PNI).

**Objective:**

To investigate the characteristics of early invasive SCC with or without PNI.

**Methods:**

Consecutive SCC excisions were prospectively reviewed from a single Australian community-based practice for 2016-2018. Tumor characteristics recorded were anatomic site, maximum microscopic tumor surface diameter, invasion depth, grade of differentiation, and diameter of nerves with PNI.

**Results:**

In total, 1,772 cases were collected. No PNI cases were found on female patients. Seven of the total 10 PNI cases were on facial sites. Maximum average microscopic tumor surface diameters ranged from 10.1 mm (well differentiated) up to 11.0 mm (moderately differentiated). Maximum average invasion depths by differentiation ranged from 1.7 (well differentiated) up to 2.6 mm (poorly differentiated). The PNI cases were as follows: well differentiated (n = 0), moderately differentiated (n = 4), or poorly differentiated (n = 6). Minimum average histopathological margins for well, moderately, and poorly differentiated SCC, respectively, were 1.4, 1.1, and 1.3 mm. Minimum microscopic tumor surface diameters for PNI cases were 7 mm for moderately and 5 mm for poorly differentiated SCC. Minimum microscopic invasion depths for PNI cases were 2.2 mm for moderate and 0.9 mm for poor differentiation.

**Conclusions:**

We found early SCC with PNI displayed nerve diameters of 0.1 mm or less and were exclusively on male patients aged 60 or older, on chronically sun-exposed sites of the head and upper midline anterior chest. Histopathological features associated with PNI were moderate and poor differentiation, tumor invasion beyond 0.9 mm, and adjacent lymphocytosis.

## Introduction

Over recent decades the prevalence of cutaneous squamous cell carcinoma (SCC) has increased [1]. A recent review [2] found multiple studies demonstrating perineural invasion (PNI) is significantly associated with a higher risk of recurrence following surgery, metastasis, and disease-specific death. Reported presentations of SCC with PNI vary from 2.5% to 6% [3]. Most studies on PNI tend to focus on advanced disease treated in tertiary facilities. PNI has been referred to as either “incidental” when asymptomatic and involving small-diameter nerves compared with “clinical” when symptomatic and involving larger nerve diameters [4]. This small nerve diameter has been defined as equal to or less than 0.1 mm [5]. Little is known of the characteristics of early-stage, smaller-dimension SCC with early-onset PNI. Over the past 2 decades, publications [2,5] have consistently stated that an increase in nerve diameters beyond 0.1 mm is associated with a poorer prognosis including disease-specific death.

Both the clinical and dermoscopic features of SCC vary with the grade of differentiation [6]. Pain and numbness have been reported as clues to PNI [7,8]. Poorly differentiated SCC tends to have a higher prevalence of PNI [9,10] compared with well- and moderately differentiated tumors. An increase in PNI has also been recorded in SCC with a desmoplastic stroma [11]. Increasing SCC tumor diameter correlates with increased depth of PNI [12]. A minimum biopsy depth of 3 to 4 mm has been recommended to optimize not missing PNI [12]. Some SCC without PNI may display features mimicking PNI [13]. Peritumoral fibrosis is a known mimic of PNI [14]. Combining epithelial membrane antigen with cytokeratin MNF116 has been stated to facilitate diagnosing PNI when both of these immunohistochemical stains are positive [15].

As PNI develops, it is not clear whether advanced PNI correlates with an increase in affected nerve diameters alone and/or with an increase in the spatial distribution of smaller affected nerve diameters. The intention of our study was to identify clinical and histopathological characteristics of early-stage SCC associated with early-appearance PNI. Identifying clinical characteristics of SCC associated with the threshold onset of early PNI may facilitate the bedside earlier detection of PNI, thus improving patient outcomes. Furthermore, an awareness of a higher chance for PNI being present based on the histopathological characteristics of an SCC case may also assist histopathologists in the early detection of PNI.

## Materials and Methods

Consecutive invasive SCC cases were prospectively collected from routine workflow in a community-based private practice in Sydney, Australia, from 2016 to 2018. The University of Queensland (Brisbane, Australia) authorized ethics approval (project no. 2016001221). Full excision was attempted on all cases by typically using a fusiform ellipse cut down to the subcutis. Cases with prior partial biopsies and known recurrent SCC cases as well as SCC receiving prior pharmaceutical or ablative treatments were excluded. All cases excised during the study were marked on a body map. This map was checked prior to subsequent excisions to exclude recurrent cases. There were no exclusions based on age, anatomic site, or nerve diameters with PNI.

Prior to excision, every case was examined using a Delta 20 T Heine dermatoscope to identify the surface margin of the tumor. Excisions were marked out with an in vivo surface margin of 1 to 2 mm. Excised tissue was sectioned by transverse bread loafing at 3-mm intervals along the entire specimen. Following H&E staining, all cases underwent histopathological examination. All cases of keratoacanthoma, SCC in situ, and any diagnosis other than invasive SCC were excluded. Depth of invasion was measured from the granular layer in the epidermis down to the deepest tumor cells to the nearest 0.1 mm. This invasion depth measurement was consistently performed independent from the adjacent skin surface. Assessing the grade of differentiation was based on the original description by Broders [16]. Where the grade of differentiation varied within a tumor mass, the grade present closest to poor differentiation was recorded for that case.

We defined PNI as malignant squamous cells identified within the perineural space that must be located outside the tumor mass. Cases with malignant cells in the perineural space where the involved nerve was only found inside the tumor mass were excluded. Two board-certified dermatohistopathologists blind to each other’s findings had to agree PNI was present within a case for PNI classification. Cases in which only 1 of the 2 histopathologists identified PNI were excluded from PNI allocation. Mimics of PNI were well known to the reporting histopathologists. To assess whether localized lymphocytosis is a clue to PNI, the co-location of lymphocytes with PNI or juxtaposed to the PNI was recorded when seen. The qualitative density of lymphocytes per unit area juxtaposed to the PNI was compared with the lymphocyte density in the background tissue well away from the involved nerve.

## Results

After initial exclusion criteria were applied, a total of 1,772 SCC cases were collected. Male patients numbered 740 (63.4%) and females 427 (36.6%). Patients had predominantly northern European ancestry with fair skin. The characteristics of the study cases are shown in Table 1. Intratumor PNI was reported in 18 cases (11 cases with moderate differentiation and 7 cases with poor differentiation). The cases with only intratumor PNI were excluded from the study. Only cases with PNI outside the tumor mass (n = 10) entered the final study results. Of these 10 PNI cases, 2 cases were noted to have both intratumor PNI and PNI outside the tumor mass.

**Figure 1:**
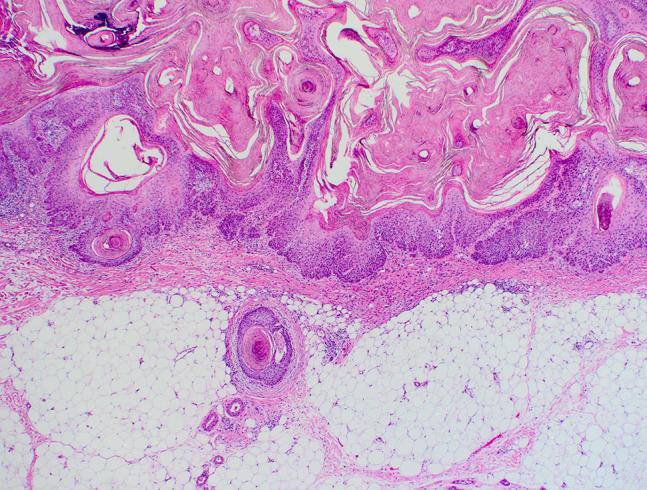
Moderately differentiated squamous cell carcinoma: H&E staining demonstrating perineural invasion displayed with magnification ×10.

**Figure 2:**
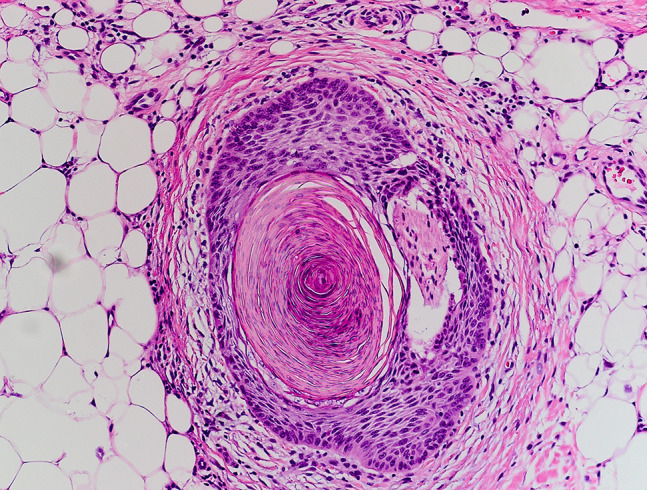
Perineural invasion below an invasive squamous cell carcinoma: same case and staining as Figure 1 at magnification ×40.

Poorly differentiated SCC cases presented with deeper average invasion (2.3 mm) and a higher prevalence of PNI cases (n = 6/10). All cases without PNI combined had an average invasion depth of 1.8 mm (95% confidence interval [1.7–1.9] P < 0.001) compared with 3.0 mm average invasion depth for all cases with PNI (95% confidence interval [1.5–4.4] P < 0.001). A minimum depth of invasion for all PNI cases by grade of differentiation was 2.2 mm for moderately and 0.9 mm for poorly differentiated cases. Moderately differentiated cases with PNI had an average invasion depth of 3.7 mm. In comparison, the average invasion depth for poorly differentiated PNI cases was 2.4 mm. An example of an invasive SCC displaying moderate differentiation and PNI is shown in Figures 1 and 2.

Although the intention was to fully excise all cases, histopathologically identified margin involvement with invasive SCC did occur in some cases. By grade of differentiation, clear histopathological margins were as follows: well n = 1,178/1,253 (94%), moderate n = 392/436 (90%), and poor differentiation n=65/83 (78%) (Table 2). Table 2 illustrates the shift from well to poor differentiation was also associated with increasing rates of peripheral only, deep only, and combined peripheral and deep margin involvement.

Nine of the 10 PNI cases recorded a nerve diameter of less than 0.1 mm (Table 3). The largest nerve diameter recorded was 0.1 mm (1 case), indicating all the study cases represent incidental PNI. The minimum ages for moderately and poorly differentiated cases, respectively, were 64 and 60 years. All 8 PNI cases not invading the subcutis displayed tumor-associated lymphocytes. Only 2 cases recorded both tumor invasion and PNI involving the subcutis. In both cases, no tumor-associated lymphocytes were found (Table 3). Table 3 also shows the anatomic sites for all PNI cases.

During the study the number of cases of SCC excised per patient was recorded and is shown in Table 4. A trend is noted where an increase in the number of SCC cases per patient during the study was associated with a higher prevalence of PNI (Table 4). Most of the PNI cases (6/10) were found on patients with 4 or more SCC cases.

## Limitations

Poorly differentiated cases had a higher rate of involved microscopic margins. As PNI was defined as being outside the tumor mass, some PNI may have be missed with involved tumor margins. Thus PNI may be underrepresented within the total poorly differentiated cases.

Host immunosuppression may be a confounding factor in the study findings. One of the moderately differentiated SCC cases had background rheumatoid arthritis of 11 years’ duration. One patient with a poorly differentiated case had chronic lymphatic leukemia known for 3 years.

## Discussion

With only 10 PNI cases in a total cohort of 1,772 cases, the overall PNI presentation was approximately 0.56%. Compared with other studies, this percentage of cases with PNI is very low. Previous PNI studies have typically been on cohorts based in tertiary facilities. There are at least 2 factors that may account for low PNI percentage in our study. First, the study deliberately selected a community-based cohort with early disease and smaller-size cases. The intention of the study was to identify whether thresholds exist for the case characteristics examined when early PNI first appears. Second, we found no cases of early PNI in well-differentiated cases. Small-dimension, well-differentiated cases may be overrepresented in our study cohort. Such cases are usually treated in community practice and do not need referral to tertiary facilities.

One striking feature of our study was that all PNI cases were on male patients. This was recorded from 1,320 male cases even though there were 452 female cases in the study. Tumor microscopic surface diameters were very similar for the 3 grades of differentiation. Threshold microscopic surface tumor diameters for the detection of PNI were 7 mm for moderately and 5 mm for poorly differentiated SCC. Tumor invasion depths progressively increased in the shift to poor differentiation, consistent with previous studies [15]. All recorded cases of PNI were either on the face (n = 7/10) or anterior upper midline chest (n = 3/10). Two of the 3 PNI cases on the chest occurred on the sun-exposed area over the manubrium. Together the anatomic sites for all 10 PNI cases displayed chronic solar damage on typically fair Caucasian skin. This suggests chronic sun exposure is a strong driver for PNI.

Head and neck have been previously reported as favored sites for PNI [9,10,12]. Multiple studies have recorded increased PNI with the shift from well to poor differentiation [9,10]. However, 2 studies [10,17] have found more cases of PNI in moderate compared with poor differentiation cases in their study cohorts. Our study found that 0.92% of moderately differentiated SCC had incidental PNI compared with 7.2% of poorly differentiated SCC with incidental PNI. When examining all cases combined, poorly differentiated SCC recorded the deepest average invasion depth. However, we found the average invasion depth of poorly differentiated SCC with PNI was just 2.4 mm compared with 3.7 mm for PNI cases with moderate differentiation. Due to a higher proportion of poorly differentiated cases displaying involved microscopic margins, it would not be surprising if the actual percentage of poorly differentiated cases with PNI was higher than recorded.

We found that 2 of the 10 PNI cases did not display tumor-associated lymphocytes when tumor invasion and PNI presence were both identified in the subcutis. The other 8 cases all displayed tumor-associated lymphocytes when tumor invasion and PNI did not extend into the subcutis (Table 3). Examination of Table 4 reveals that patients with 4 or more SCC cases were more likely to have PNI.

A single case of acantholytic SCC was noted in 1 of the 10 cases with PNI. This case occurred on the cheek of a man aged 75 years with an invasion depth of only 0.9 mm. This case sounds a warning that early SCC with poor differentiation and an invasion depth of only 1 mm may present with PNI. For moderately differentiated SCC we found the threshold for increased vigilance for PNI was an invasion depth of 2.2 mm. The relatively uncommon prevalence of both acantholysis and PNI limits investigation of their potential association.

Lymphovascular invasion was also found in another single PNI case. This case occurred on the eyelid of a man aged 80 years with an SCC displaying infiltrative architecture, multifocal PNI, and an invasion depth of just 2.4 mm. Whether there is an association between infiltrative architecture or desmoplasia and early multifocal PNI may be worth further study.

Metastatic behavior from a primary SCC with an invasion depth of less than 2 mm and desmoplastic histology has been reported [18]. However, overall metastatic potential in SCC requires an invasion depth to exceed 2 mm [18]. As invasion depth increases beyond 2 mm so does the increase in metastatic potential. We found deeper tumor invasion of 2.2 mm or greater was also associated with increasing prevalence of PNI. Histologically identified solar elastosis was an anecdotal observation with most PNI cases in our study. The extent to which solar elastosis is a marker for PNI risk also invites future investigation.

## Conclusions

Our study found early incidental PNI on men aged 60 years or older with a high prevalence on facial sites and patients with four or more SCC cases. Threshold histopathological features associated with early PNI included increased surface microscopic diameters greater than 5.0 mm, tumor invasion depth beyond 0.9 mm, either moderate or poor differentiation, and adjacent lymphocytosis.

## Figures and Tables

**Table 1:** Squamous Cell Carcinoma: Characteristics of Study Cases by Grade of Differentiation

	Well (n = 1,253)	Moderate (n = 436)	Poor (n = 83)
Age (male n = 1,319)			
Mean	73	74	73
IQR	66–81	67–82	64–83
n	897	358	65
Age (female n = 452)			
Mean	72	74	81
IQR	65–80	67–83	70–91
n	356	78	18
Maximum microscopic average surface diameter (mm)			
Diameter	10.1	11.0	10.7
P	<0.0001	<0.0001	0.048
IQR	7–13	8–14	7–14
Maximum average invasion depth (mm)			
Depth	1.7	2.1	2.6
P	<0.0001	<0.0001	<0.0001
IQR	1–2.1	1.3–2.5	1.1–3.0
Perineural invasion present (all male)			
n	0	4 (0.92%)	6 (7.2%)

IQR = interquartile range.

**Table 2:** Squamous Cell Carcinoma: Recorded Margin Involvement by Grade of Differentiation

Grade of Differentiation	Well (n = 1,253)	Moderate (n = 436)	Poor (n = 83)

Clear margins	n = 1,178 (94%)	n = 392 (90%)	n = 65 (78%)

Peripheral margin only involved	n = 38 (3%)	n = 22 (5%)	n = 5 (6%)

Deep margin only involved	n = 12 (1%)	n = 9 (2%)	n = 7 (9%)
Peripheral and deep margin both involved	n = 25 (2%)	n = 13 (3%)	n = 6 (7%)

Peripheral only involved margin, deep only involved margin, and both peripheral and deep margins involved are separately listed.

**Table 3:** Squamous Cell Carcinoma With Perineural Invasion (PNI): Characteristics of All Cases Found in the Study

Patient All Male	Age (yrs)	Site	Grade of Differentiation	Tumor Surface Diameter (mm)	Tumor Invasion Depth (mm)	PNI Nerve Diameter (mm)	Tumor in Subcutis (Yes/No)	PNI in Subcutis (Yes/No)	Tumor Associated Lymphocytes (Yes/No)
1	60	Forehead	Poor	11	2.2	0.04	N	N	Y
2	64	Shoulder	Mod	23	7.7	0.04	Y	Y	N
3	69	Chest	Poor	8	2.3	0.03	Y	N	Y
4	71	Chest	Mod	12	2.5	0.05	N	N	Y
5	75	Cheek	Poor	8	0.9	0.07	N	N	Y
6	80	Forehead	Poor	10	3.0	0.03	N	N	Y
7	85	Cheek	Mod	15	2.2	0.03	N	N	Y
8	87	Cheek	Mod	7	2.2	0.05	N	N	Y
9	80	Eyelid	Poor	5	2.4	0.1	Y	Y	N
10	81	Cheek	Poor	11	3.6	0.07	N	N	Y

**Table 4:** Squamous Cell Carcinoma (SCC) With Perineural Invasion (PNI): Patients by Number of SCC Cases

	n	Patients With Only 1 SCC Case	Patients With 2 SCC Cases	Patients With 3 SCC Cases	Patients With >3 SCC Cases
No. of patients (no PNI)	1,157	745 (64.4%)	229 (19.8%)	67 (5.8%)	116 (10.0%)
No. of patients (with PNI)	10	3	1	0	6

The number “n” lists the number of patients with squamous cell carcinoma, not the number of SCC cases.
